# Risk factors for microbiological persistence after 6 months of treatment for *Mycobacterium intracellulare* and its impact on the drug-resistance profile

**DOI:** 10.1128/spectrum.00805-23

**Published:** 2023-09-25

**Authors:** Xuejiao Luo, Xubin Zheng, Yong Fang, Fangyou Yu, Haiyan Cui, Qin Sun, Wei Sha

**Affiliations:** 1 Department of Tuberculosis, Shanghai Pulmonary Hospital, School of Medicine, Tongji University, Shanghai, China; 2 Clinical and Research Center for Tuberculosis, Shanghai Key Laboratory of Tuberculosis, Shanghai Pulmonary Hospital, School of Medicine, Tongji University, Shanghai, China; 3 Department of Clinical Laboratory, Shanghai Pulmonary Hospital, School of Medicine, Tongji University, Shanghai, China; University of Southern California, Duarte, California, USA

**Keywords:** *Mycobacterium intracellulare*, minimum inhibitory concentration, clinical characteristics, nontuberculous mycobacteria, culture conversion, antimycobacterial treatment

## Abstract

Patients with *Mycobacterium intracellulare* pulmonary disease are more likely to experience poor treatment outcomes if they have been observed with microbiological persistence after 6 months of treatment. This study aims to identify the risk factors for microbiological persistence and describe the changes in the minimum inhibitory concentration (MIC) during antimycobacterial treatment. This retrospective case-control study enrolled patients diagnosed with *M. intracellulare* pulmonary disease between April 2017 and September 2021 at Shanghai Pulmonary Hospital. Patients with positive cultures after 6 months of treatment (positive group) were matched by age and sex in a 1:1 ratio to patients with negative conversion (negative group). Totally, 46 pairs of patients were analyzed. Risk factors for microbiological persistence at month 6 were smoking, previous tuberculosis treatment, chronic lung diseases, a positive baseline acid-fast bacilli smear, and adverse drug reactions; the risk was reduced by a regimen containing ethambutol, ≥3 effective drugs, and a higher pre-treatment absolute lymphocyte count. Regarding the drug-resistance profile, the negative group had a higher proportion of susceptibility to clarithromycin (100.0% vs 84.8%, *P* = 0.012). Most isolates were susceptible or intermediate to amikacin in both groups (93.5% and 84.8%, respectively). Nine patients (16.4%, 9/55) had a change in the drug-resistance profile, including four who changed from clarithromycin susceptible to clarithromycin resistant, and the other three reversed. Two pairs of isolates had a change in resistance to amikacin. In conclusion, risk factors for microbiological persistence were identified, and the change in MIC values during antimycobacterial treatment indicated the need for monitoring to enable timely adjustment of the regimen.

**IMPORTANCE:**

Nontuberculous mycobacteria pulmonary disease (NTM-PD) has been recognized as an important public health issue because of its increasing incidence globally, low cure rate, and high recurrence rate. NTM-PD has innate resistance to many first-line anti-tuberculous drugs, which limits the treatment options. *Mycobacterium intracellulare* is reportedly the most important pathogenic NTM and accounts for the highest proportion of NTM-PD in China. A previous study suggested that poor microbiological response after 6 months of treatment is predictive of treatment failure. The present study investigated the risk factors associated with persistent positive sputum cultures by treatment month 6 in patients with *M. intracellulare* pulmonary disease and the variation in minimum inhibitory concentration patterns in clinical settings. This information might help to identify patients at higher risk of treatment failure and enable the timely provision of necessary interventions.

## INTRODUCTION

The incidence and prevalence of nontuberculous mycobacteria pulmonary diseases (NTM-PD) are increasing worldwide (1 – 4). A national epidemiological survey showed that the percentage of NTM strains among mycobacterial isolates in China has markedly increased from 11.1% in 1990 to 22.9% in 2010 (5). The most common pathogen causing NTM-PD is *Mycobacterium avium complex* (MAC), mainly composed of *M. avium* and *M. intracellulare*. However, the treatment for MAC pulmonary disease is challenging due to the long treatment duration, need for multiple antibiotics, high incidence of drug-induced adverse events, and poor treatment response (6, 7). *M. intracellulare* alone reportedly causes more than 50% of NTM-PD in China (8) and results in more severe clinical characteristics and a worse prognosis than *M. avium* (9). Up to 40% of patients with *M. intracellulare* pulmonary disease fail to respond to long-term chemotherapy or develop relapse or reinfection soon after initial treatment success (6). Even worse, some patients finally develop refractory MAC pulmonary disease, for which the treatment options are very limited.

Long-term administration of antibiotics is important in treating MAC. The clinical practice guidelines of the American Thoracic Society (ATS), European Respiratory Society (ERS), European Society of Clinical Microbiology and Infectious Diseases (ESCMID), and Infectious Disease Society of America (IDSA) recommend the administration of antibiotics for a minimum of 12 months after negative culture conversion (10). Recent studies found that the factors related to culture conversion in patients with MAC pulmonary disease are radiological type, old age, and time to culture positivity at baseline (11 – 13). The Clinical and Laboratory Standards Institute (CLSI) guidelines recommend drug susceptibility testing (DST) for macrolides and amikacin to guide the design of the treatment regimen and optimize the treatment response (14 – 16). Notably, the acquisition of drug resistance during treatment always indicates a poor treatment response and increases the risk of mortality, consistent with the findings for multidrug-resistant tuberculosis (TB) (17). Therefore, it is essential to monitor the drug-resistance profile during the long-term use of antibiotics. To our knowledge, there have been two studies for patients with *Mycobacterium abscessus* and *Mycobacterium kansasii* pulmonary disease, which have focused on the change in the drug-resistance profile before and after antibacterial treatment (18, 19). However, this information is still lacking for MAC pulmonary disease.

The present study aims to describe the distributions and dynamic changes in the minimum inhibitory concentration (MIC) for the key drugs used in the antimycobacterial treatment and to identify the risk factors for failed sputum culture conversion by treatment month 6 in patients with *M*. *intracellulare* pulmonary disease.

## RESULTS

### Patient characteristics

**Fig 1:**
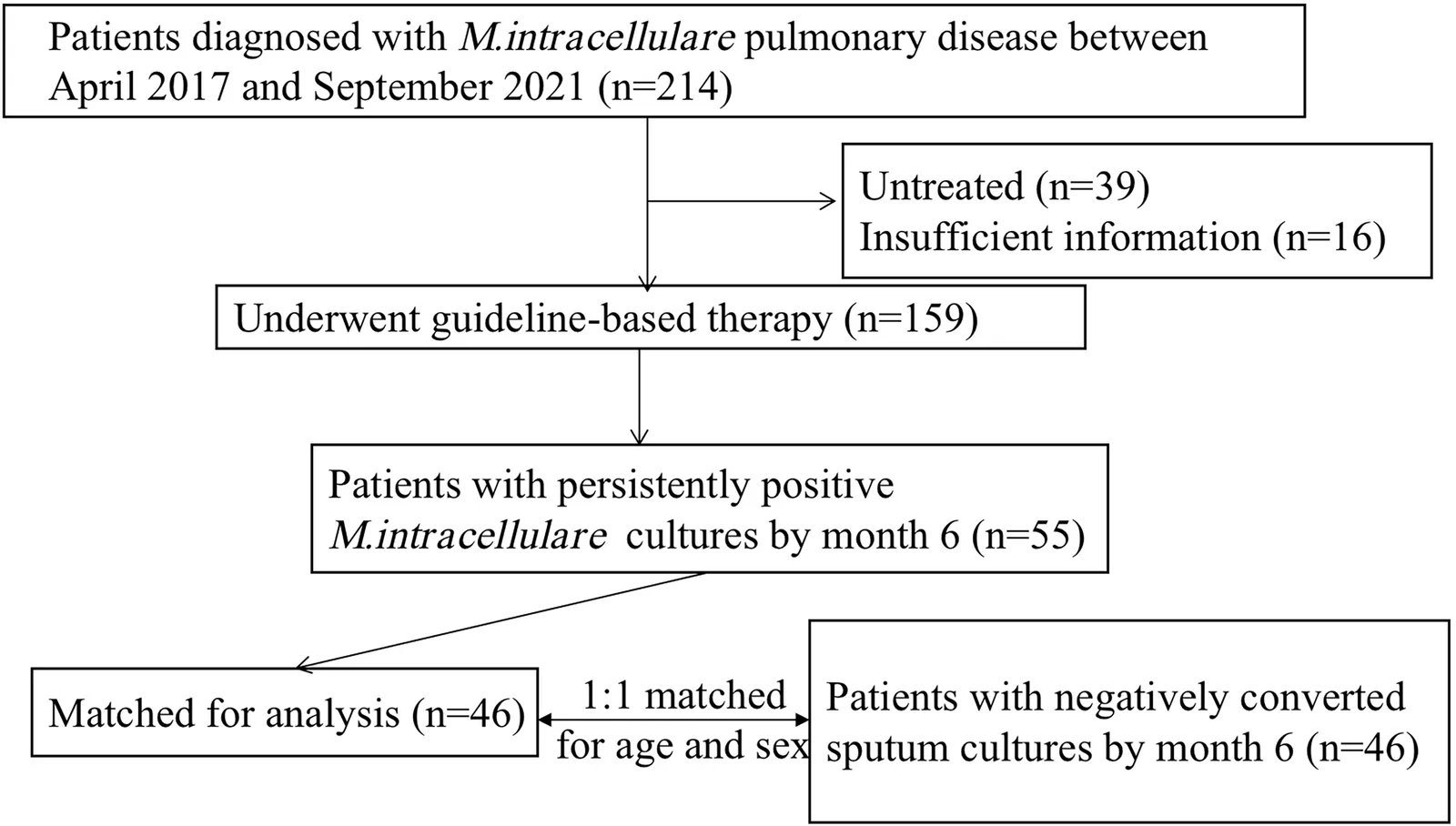
Flowchart for enrollment of patients with *Mycobacterium intracellulare* pulmonary disease.

A total of 55 patients with *M. intracellulare* pulmonary disease had a persistent positive culture after 6 months of treatment during the study period and were included in the positive group. After 1:1 matching for age and sex with patients with a negative culture at treatment month 6 (negative group), 46 pairs of patients were included for analysis (Fig. 1). The patients in the positive group who failed to be matched were mainly those aged above 80 years. Compared to the negative group, the positive group had a lower body mass index (18.1 vs 20.5 kg/m^2^, *P* = 0.006) and a higher prevalence of previous TB treatment (28% vs 14%, *P* = 0.003); the positive group also had a significantly higher prevalence of smoking (15% vs 2%, *P* < 0.001), chronic lung disease (52.2% vs 23.9%, *P* = 0.005), and a positive acid-fast bacilli (AFB) smear at baseline (80.4% vs 47.8%, *P* = 0.001). The radiological presentations varied between groups (*P* < 0.001). In particular, the negative group had a higher prevalence of the noncavitary nodular-bronchiectatic (NC-NB) form (73.9% vs 26.1%), whereas the positive group had a higher prevalence of the cavitary nodular-bronchiectatic (C-NB) (41.3% vs 21.7%) and fibrocavitary (FC) forms (32.6% vs 4.3%) (Table 1).

**TABLE 1:** Baseline characteristics for patients with *Mycobacterium intracelluare* pulmonary disease in this study^*a*^

Characteristics^*b*^	Positive group (*n* = 46)	Negative group (*n* = 46)	Total (*n* = 92)	*P* value
Age (years)	61.4 ± 9.2	60.8 ± 8.5	61.1 ± 8.8	0.752
Female	31 (67.4)	31 (67.4)	62 (67.4)	1.000
BMI (kg/m^2^)	18.1 (16.2, 20.5)	20.5 (18.7, 23.1)	19.4 (17.3, 22.5)	0.006
Smoking	15 (32.6)	2 (4.3)	17 (18.5)	<0.001
Previous TB treatment history	28 (60.9)	14 (30.4)	42 (45.7)	0.003
Symptoms				
Cough and expectoration	45 (97.8)	41 (89.1)	86 (93.5)	0.203
Hemoptysis	15 (32.6)	9 (19.6)	24 (26.1)	0.154
Radiographic findings				
NC-NB	12 (26.1)	34 (73.9)	46 (50.0)	<0.001
C-NB	19 (41.3)	10 (21.7)	29 (31.5)	
FC	15 (32.6)	2 (4.3)	17 (18.5)	
Comorbidity				
Hypertension	3 (6.5)	6 (13.0)	9 (9.8)	0.485
Diabetes type 2	1 (2.2)	3 (6.5)	4 (4.3)	0.617
Chronic lung disease	24 (52.2)	11 (23.9)	35 (38.0)	0.005
Malignant tumor	4 (8.7)	1 (2.2)	5 (5.4)	0.361
Rheumatism	7 (15.2)	2 (4.3)	9 (9.8)	0.158
Positive AFB smear at baseline	37 (80.4)	22 (47.8)	59 (64.1)	0.001
Laboratory tests				
ESR (mm/h)	62.5 (27.0, 95.5)	24.5 (11, 35.5)	32.0 (20.0, 70.0)	<0.001
Lymphocyte count (×10^9^/L)	1.2 (1.0, 1.5)	1.4 (1.1, 1.7)	1.3 (1.0, 1.5)	0.001
CD4^+^ T cell (%)	31.1 (24.7, 39.8)	38.8 (33.7, 43.1)	36.3 (28.8, 41.8)	<0.001
CD8^+^ T cell (%)	18.1 (14.2, 29.2)	16.1 (14, 20.1)	17.0 (14.3, 22.9)	0.116
CD4/CD8 T-cell ratio	1.5 (0.9, 2.4)	2.2 (1.9, 2.8)	2.1 (1.3, 2.6)	0.001
Drug intake				
Macrolides	42 (91.3)	46 (100.0)	88 (95.7)	0.117
Ethambutol	29 (63.0)	42 (91.3)	71 (77.2)	0.001
Rifampicin	32 (69.6)	42 (91.3)	74 (80.4)	0.009
Rifabutin	5 (10.9)	3 (6.5)	8 (8.7)	0.714
Amikacin	13 (28.3)	19 (41.3)	32 (34.8)	0.189
Moxifloxacin	11 (23.9)	25 (54.3)	36 (39.1)	0.003
Levofloxacin	12 (26.1)	7 (15.2)	19 (20.7)	0.198
Number of effective drugs				
<3	17 (37.0)	3 (6.5)	20 (21.7)	<0.001
≥3	29 (63.0)	43 (93.5)	72 (78.3)	
Adverse drug reactions	30 (65.2)	21 (45.7)	51 (55.4)	0.059
Gastrointestinal reaction	10 (21.7)	6 (13.0)	16 (17.4)	0.271
Liver injury	8 (17.4)	3 (6.5)	11 (12.0)	0.108
Drug allergy	3 (6.5)	4 (8.7)	7 (7.6)	1.000
Hematological toxicity	2 (4.3)	5 (10.9)	7 (7.6)	0.434
Neurotoxicity	5 (10.9)	3 (6.5)	8 (8.7)	0.714
Others	2 (4.3)	0 (0.0)	2 (2.2)	0.495
Regimen adjustment	27 (58.7)	16 (34.8)	43 (46.7)	0.022
Treatment interruption	8 (17.4)	1 (2.2)	9 (9.8)	0.030

^*a*^ All data are shown as proportions for categorical variables; as mean ± SD for normally distributed data or as median with IQR for nonnormally distributed continuous variables.

^*b*^ BMI, body mass index; TB, tuberculosis; NC-NB, noncavitary nodular bronchiectatic; C-NB, cavitary nodular bronchiectatic; FC, fibrocavitary; AFB, acid-fast bacilli; ESR, erythrocyte sedimentation rate.

In total, 51 patients once experienced adverse drug reactions during the first 6 months of treatment and 84.3% of them followed by regimen adjustment, including change of drug regimen or lowering of doses. Compared to the negative group, adverse drug reactions were more commonly seen in the positive group (65.2% vs 45.7%, *P* = 0.059) (Table 1). Specifically, the incidences of gastrointestinal reaction (21.7% vs 13.0%, *P* = 0.271), liver injury (17.4% vs 6.5%, *P* = 0.108), and neurotoxicity (10.9% vs 6.5%, *P* = 0.714) were higher in the positive group. Nine patients had once interrupted their treatment during the first 6 months, with eight in the positive group and one in the negative group. Eight of them were due to adverse drug reactions and interrupted for 1 to 2 weeks, while the other one was due to poor adherence. The incidence of treatment interruption was significantly higher in the positive group than in the negative group (17.4% vs 2.2%, *P* = 0.030).

### Treatment regimens

Macrolides were the most frequently used drugs (95.7%), followed by rifampin (80.4%), ethambutol (77.2%), moxifloxacin (39.1%), and amikacin (34.8%) (Table 1). The positive group had a higher incidence of regimen adjustment than the negative group (58.7% vs 34.8%, *P* = 0.022). Compared to the positive group, a higher proportion of patients in the negative group received ethambutol (91.3% vs 63.0%), rifampin (91.3% vs 69.6%), and moxifloxacin (54.3% vs 23.9%) (all *P* < 0.01). The number of administered drugs was two or less for 37% of patients in the positive group and 6.5% in the negative group (*P* < 0.001) (Table 1).

### Risk factors for 6-month sputum culture results

As shown in Table 2, multivariable analyses showed that the factors independently associated with an increased risk of persistent positive culture by month 6 were smoking [adjusted odds ratio (aOR), 15.9; 95% confidence interval (CI), 1.97–128.2], previous TB treatment (aOR, 4.10; 95% CI, 1.49–11.3), chronic lung disease (aOR, 4.67; 95% CI, 1.45–15.1), positive AFB smear at baseline (aOR, 4.84; 95% CI, 1.65–14.2), adverse drug reactions (aOR, 1.93; 95% CI, 0.86–4.31), treatment regimen adjustment (aOR, 2.17; 95% CI, 0.95–4.94), and treatment interruption (aOR, 7.64; 95% CI, 0.91–64.0). Compared to the patients with the NC-NB form of disease, those with the C-NB (aOR, 2.92; 95% CI, 0.94–9.08) and FC (aOR, 13.7; 95% CI, 2.48–76.0) forms had a significantly higher risk of failing to achieve negative culture conversion at 6 months. In contrast, the factors negatively associated with a positive 6-month sputum culture were a regimen containing ethambutol (aOR, 0.26; 95% CI, 0.08– 0.79), administration of three or more effective drugs (aOR, 0.08; 95% CI, 0.01–0.64), a higher absolute lymphocyte count at baseline (aOR, 0.11; 95% CI, 0.03–0.46), CD4^+^ T cell (aOR, 0.90; 95% CI, 0.83–0.97), and CD4/CD8 T-cell ratio (aOR, 0.61; 95% CI, 0.38–0.99) (Table 2).

**TABLE 2:** Univariable and multivariable analyses for risk factors associated with persistent positive sputum culture after 6-month antimycobacterial treatment

Variable	Positive group (*n* = 46)	Negative group (*n* = 46)	OR (95% CI)	aOR (95% CI)
Smoking	15 (32.6)	2 (4.3)	14.0 (1.84,106.5)	15.9 (1.97, 128.2)
Previous TB treatment history	28 (60.9)	14 (30.4)	3.00 (1.28, 7.06)	4.10 (1.49, 11.3)
Radiographic findings				
NC-NB^*a*^	12 (26.1)	32 (69.6)	1	1
C-NB	19 (41.3)	10 (21.7)	3.75 (1.29, 10.9)	2.92 (0.94, 9.08)
FC	15 (32.6)	2 (4.3)	10.5 (2.23, 49.8)	13.7 (2.48, 76.0)
Chronic lung disease	24 (52.2)	11 (23.9)	4.25 (1.43, 12.6)	4.67 (1.45, 15.1)
Positive AFB smear at baseline	37 (80.4)	22 (47.8)	4.00 (1.50, 10.7)	4.84 (1.65, 14.2)
Laboratory tests				
ESR (mm/h)	62.5 (27.0, 95.5)	24.5 (11, 35.5)	1.03 (1.01, 1.05)	1.03 (1.01, 1.04)
Lymphocyte count (×10^9^/L)	1.2 (1.0, 1.5)	1.4 (1.1, 1.7)	0.12 (0.03, 0.47)	0.11 (0.03, 0.46)
CD4^+^ T cell (%)	31.1 (24.7, 39.8)	38.8 (33.7, 43.1)	0.89 (0.83, 0.96)	0.90 (0.83, 0.97)
CD4/CD8 T-cell ratio	1.5 (0.9, 2.4)	2.2 (1.9, 2.8)	0.56 (0.35, 0.90)	0.61 (0.38, 0.99)
Drug intake				
Ethambutol	29 (63.0)	42 (91.3)	0.24 (0.08, 0.70)	0.26 (0.08, 0.79)
Rifamycin	32 (69.6)	42 (91.3)	0.11 (0.01, 0.88)	0.14 (0.02, 1.17)
Amikacin	13 (28.3)	19 (41.3)	0.54 (0.22, 1.35)	0.68 (0.26, 1.80)
Number of effective drugs				
<3	17 (37.0)	3 (6.5)	1	1
≥3	29 (63.0)	43 (93.5)	0.07 (0.01, 0.51)	0.08 (0.01, 0.64)
Adverse drug reactions	30 (65.2)	21 (45.7)	1.90 (0.88, 4.09)	1.93 (0.86, 4.31)
Regimen adjustment	27 (58.7)	16 (34.8)	2.22 (1.01, 4.88)	2.17 (0.95, 4.94)
Treatment interruption	8 (17.4)	1 (2.2)	8.00 (1.00, 64.0)	7.64 (0.91, 64.0)

^*a*^ aOR, adjusted odds ratio; 95% CI, 95% confidence interval; TB, tuberculosis; NC-NB, noncavitary nodular bronchiectatic; C-NB, cavitary nodular bronchiectatic; FC, fibrocavitary; AFB, acid-fast bacilli; ESR, erythrocyte sedimentation rate.

### Drug-resistance profile at baseline

**Fig 2:**
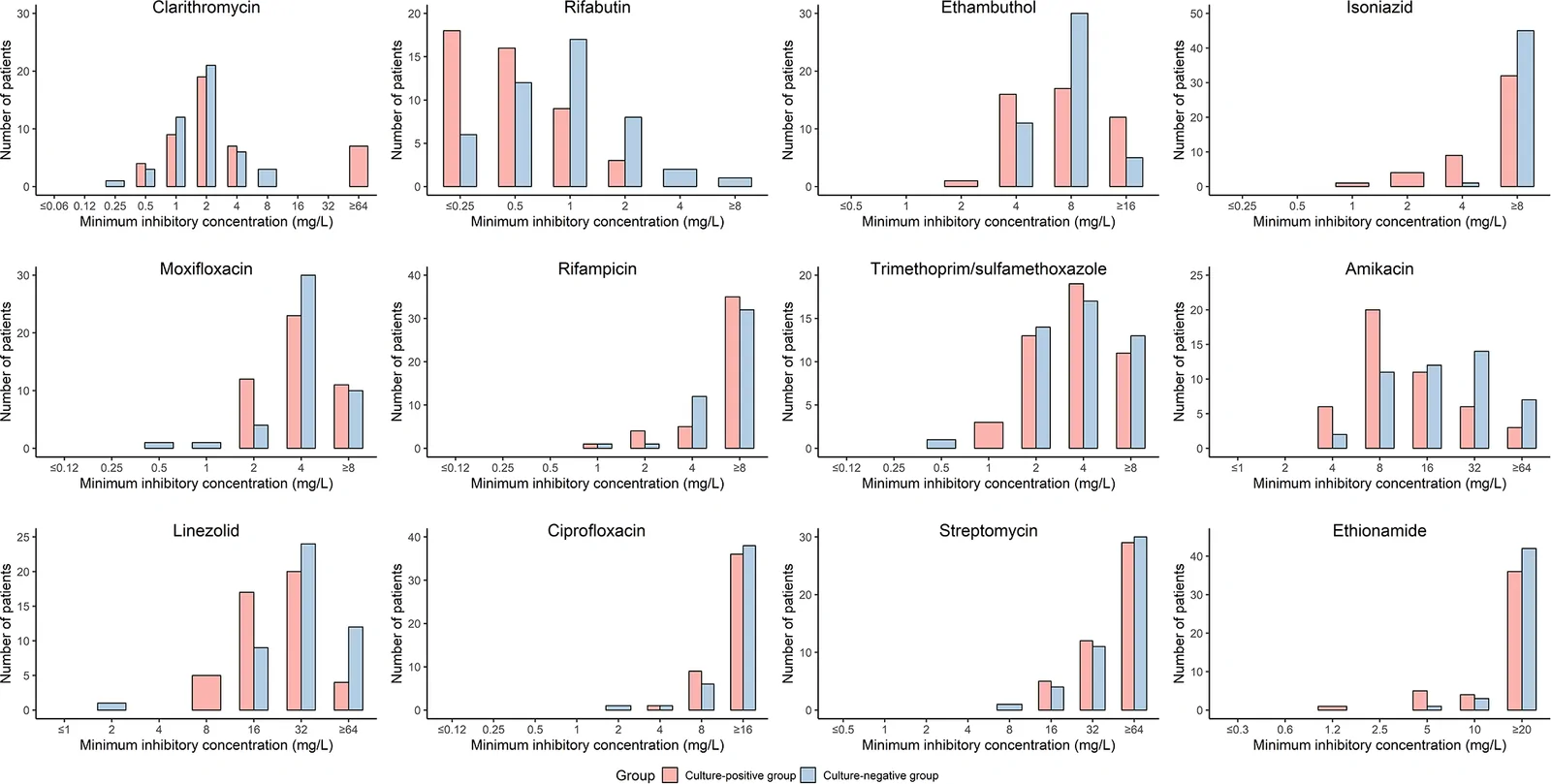
Distribution of MIC for 12 drugs in patients with *Mycobacterium intracellulare* pulmonary disease. The MIC values for doxycycline were not presented because 98% of them were >16 mg/L.

Thirty-nine isolates (84.8%) in the positive group were susceptible to clarithromycin, whereas all isolates in the negative group were susceptible to clarithromycin (*P* = 0.012) (Table 3). Most isolates were susceptible or intermediate to amikacin in both the negative and positive groups (93.5% and 84.8%, respectively), and over half were resistant to linezolid (52.2% and 78.3%, respectively) and moxifloxacin (73.9% and 87.0%, respectively). The proportion of isolates with rifabutin MIC values below 1 mg/L was lower in the negative group than the positive group (76% vs 93%, *P* = 0.002) (Fig. 2). In addition, 69 (75%) isolates had MIC values below 2 mg/L for clarithromycin and 62 (67%) isolates had amikacin MIC values below 16 mg/L. Only two isolates (2%) were susceptible to moxifloxacin with MIC values less than 1 mg/L. The rifabutin MIC values were lower than the rifampin MIC values (MIC_50_ 0.5 vs 8 mg/L) (Fig. 2).

**TABLE 3:** Drug-resistance profile for clarithromycin, amikacin, moxifloxacin, and linezolid in clinical *Mycobacterium intracellulare* isolates

Antimicrobial agent	Positive group (*n* = 46)	Negative group (*n* = 46)	Total (*n* = 92)	*P* value
Clarithromycin				
Susceptible or intermediate	39 (84.8)	46 (100.0)	85(92.4)	0.012
Susceptible (≤8)	39 (84.8)	46 (100.0)	85(92.4)	
Intermediate (=16)	0 (0.0)	0 (0.0)	0 (0.0)	
Resistant (≥32)	7 (15.2)	0 (0.0)	7 (7.6)	
Amikacin				
Susceptible or intermediate	43 (93.5)	39 (84.8)	82 (89.1)	0.180
Susceptible (≤16)	37 (80.4)	25 (54.3)	62 (67.4)	
Intermediate (=32)	6 (13.0)	14 (30.4)	20(21.7)	
Resistant (≥64)	3 (6.5)	7 (15.2)	10 (10.9)	
Moxifloxacin				
Susceptible or intermediate	12 (26.1)	6 (13.0)	18 (19.6)	0.115
Susceptible (≤1)	0 (0.0)	2 (4.3)	2 (2.2)	
Intermediate (=2)	12 (26.1)	4 (8.7)	16 (17.4)	
Resistant (≥4)	34 (73.9)	40 (87.0)	74 (80.4)	
Linezolid				
Susceptible or intermediate	22 (47.8)	10 (21.7)	32 (34.8)	0.009
Susceptible (≤8)	5 (10.9)	1 (2.2)	6 (6.5)	
Intermediate (=16)	17 (37.0)	9 (19.6)	26 (28.3)	
Resistant (≥32)	24 (52.2)	36 (78.3)	60 (65.2)	

### Longitudinal changes in MIC after 6 months of antimycobacterial treatment

**Fig 3:**
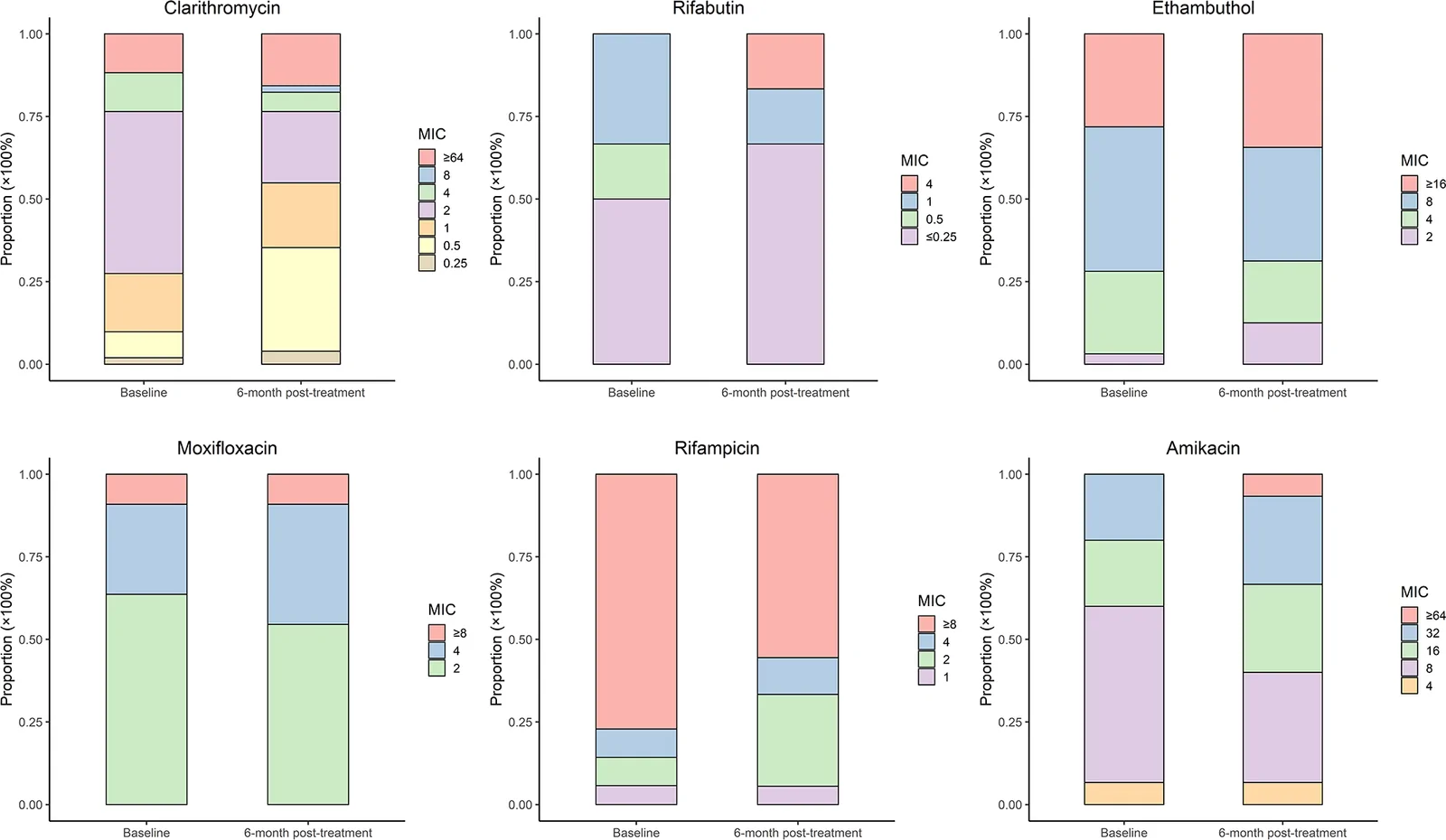
Change of MIC for key antimycobacterial drugs in patients with *Mycobacterium intracellulare* pulmonary disease before and after 6-month treatment. The number of patients who received clarithromycin, rifampicin, ethambutol, amikacin, moxifloxacin, and rifabutin was 51, 36, 32, 15, 11, and 6 patients, respectively.

Among the 55 patients in the positive group, 51 were treated with clarithromycin, followed by rifampin (*n* = 36), ethambutol (*n* = 32), amikacin (*n* = 15), moxifloxacin (*n* = 11), and rifabutin (*n* = 6). Nine pairs of isolates (16.4%) had a change in the drug-resistance profile, 88.9% (8/9) of which happened under the administration of respective drugs. In particular, four isolates changed from clarithromycin susceptible to clarithromycin resistant, while three changed from clarithromycin resistant to clarithromycin susceptible. Two pairs of isolates had a change in resistance to amikacin (one from susceptible to resistant, and one from resistant to susceptible in a patient without the intake of amikacin). Among patients taking the respective drugs, the rifampin MIC values significantly decreased (*P* = 0.031), while the MIC results for moxifloxacin and ethambutol remained stable during the 6 months of treatment (Fig. 3).

## DISCUSSION

The present study shows that smoking, previous TB treatment, chronic lung disease, a positive AFB smear before treatment, cavitary lesions, adverse drug reactions, and clarithromycin resistance were risk factors for microbiological persistence after 6 months of antimycobacterial treatment. In contrast, the probability of negative culture conversion increased in patients who were administered a regimen containing ethambutol, three or more effective drugs, and had a higher absolute lymphocyte count at the initiation of treatment. The clinical isolates had higher proportions of susceptibility to clarithromycin and amikacin than moxifloxacin. Furthermore, 16.4% of patients with microbiological persistence had obvious changes in susceptibility to clarithromycin and amikacin.

The recently published ATS/ERS/ESCMID/IDSA guidelines have well summarized the criteria for regimen design, drug doses, and treatment duration (10). However, there are still some unmet needs both for patients and physicians. Overall, the treatment and management of MAC pulmonary disease are fraught with challenges. A previous review suggested an integrated treatment approach beyond antibiotics, including avoiding exposure to environments where mycobacteria are present, implementing a personalized pulmonary rehabilitation plan and airway clearance techniques, nutritional evaluation and intervention, and managing comorbidities (20).

The present study identified risk factors for the failure of culture conversion by treatment month 6. The presence of cavitary lesions and previous anti-TB treatment increased the risk of microbiological persistence, consistent with previous studies (21, 22). One possible explanation for this finding is that high bacterial loads exist in pulmonary cavitation. Furthermore, the destroyed vascular architecture prevents the penetration of drugs into cavitary lesions (23). For this reason, the ATS/ERS/ESCMID/IDSA guidelines recommend that parenteral amikacin or streptomycin should be included in the initial treatment regimen for those with cavitary lesions. This was supported by a study that found that patients with cavitary MAC pulmonary disease have higher rates of treatment success when treated with aminoglycosides for 3 or more months (24).

It is not surprising that the baseline drug-resistance profile affects the treatment efficacy, especially for clarithromycin (25, 26). In the present study, the positive group had a lower proportion of patients with susceptibility to clarithromycin at baseline than the negative group (84.8% vs 100%). As reported in previous studies, the sputum culture conversion rate falls from approximately 80% to only 5%–36% when there is macrolide resistance (27, 28). Furthermore, amikacin was used more frequently in the negative group than the positive group (41.3% vs 28.3%). A cohort study found that amikacin must be used in combination with adequate companion medications, such as a macrolide, ethambutol, and possibly rifampin and clofazimine, to prevent the emergence of acquired drug resistance and the failure of sputum culture conversion (29). These results are consistent with our findings that a regimen containing three or more effective drugs was associated with a better treatment response. In the present study, only 2% and 6.5% of isolates were defined as susceptible to moxifloxacin and linezolid, respectively, using the tentative breakpoints from the CLSI guidelines (30); however, the limited sample size prevents analyses of the correlations with the treatment response. Thus, a larger study is needed to validate the *in vitro-in vivo* correlations for moxifloxacin and linezolid in clinical settings.

The occurrence, development, and prognosis of NTM are closely related to the immune status and immune response of the host. The absolute lymphocyte count, which includes the total number of T cells, B cells, and natural killer cells, plays a very important role in NTM infection (15, 31). Previous studies have shown that lymphopenia increases the risk of death from a variety of causes, including cancer, cardiovascular diseases, and respiratory illnesses (32). Notably, lymphopenia is also a reliable predictor of poor outcomes in patients with COVID-19 and MAC pulmonary diseases (33, 34). In our study, a higher absolute lymphocyte count at baseline was negatively associated with the presence of a positive 6-month sputum culture. Furuuchi et al. showed that posttreatment lymphopenia is associated with an increased risk of recurrence in patients with MAC pulmonary diseases (35). Additionally, Shu et al. reported that programmed cell death-1 and markers of apoptosis on lymphocytes are significantly increased in patients with MAC pulmonary diseases (36). Thus, the lymphocyte count may reflect the immune status and serve as a biomarker to predict the disease course of MAC pulmonary diseases.

Ethambutol inhibits arabinosyl transferase and blocks arabinogalactan synthesis, which form part of the mycobacterial wall (37). Furthermore, synergistic effects can occur when ethambutol is used with other antimycobacterial agents. In the current study, a treatment regimen containing ethambutol was positively associated with negative sputum culture conversion. Similar results have also been shown in other studies (38, 39). However, long-term administration of ethambutol causes adverse events such as ocular toxicity, skin rash, and gastrointestinal disturbance (40, 41). The high incidence of adverse events results in the discontinuation of ethambutol before treatment completion. A previous study confirmed that the adverse events associated with ethambutol are dose-dependent (40). Furthermore, a retrospective cohort study conducted to assess whether lower ethambutol doses impact the clinical outcomes of *M. avium* and *M. intracellulare* pulmonary disease found that an ethambutol dosage of 12.5 mg/kg of body weight/day or less in guideline-based chemotherapy may reduce the incidence of optic neuropathy without worsening clinical outcomes (42). Given the positive impact of ethambutol on microbiological cure, clinicians should continue treatment with ethambutol unless definite and serious adverse events develop.

Although rifabutin has a much lower MIC than rifampicin, its clinical efficacy is controversial. It is important to balance efficacy and tolerance when making clinical decisions. Rifabutin is recommended by the clinical practice guidelines of ATS/ERS/ESCMID/IDSA for the treatment of disseminated and refractory MAC pulmonary disease. Kim et al. reported that rifabutin has the lowest MIC values against all NTM species, including MAC, *M. abscessus*, and *M. kansasii* (43). For MAC, rifabutin has MIC_50_ (≤0.062–0.5 mg/L) and MIC_90_ (0.25–1 mg/L) values of 16–256 times lower than those for other types of rifamycin (43). Furthermore, rifabutin has strong *in vitro* activity against macrolide- and aminoglycoside-resistant NTM isolates (43). These findings suggest that it might be worth considering rifabutin as a therapeutic option for NTM disease, particularly drug-resistant disease (43). Our study also found that the rifabutin MIC values were much lower than the rifampin MIC values (MIC_50_ 0.5 vs 8 mg/L). However, a meta-analysis showed that rifampin is not inferior to rifabutin and may lead to better treatment success rates for MAC (44). This may be because rifabutin has traditionally been recommended for disseminated MAC mostly seen in patients with HIV, and the prevalence of concomitant HIV was much lower in the rifampin studies than in the rifabutin studies. Therefore, there is an urgent need for large multicenter randomized controlled trials that compare rifabutin and rifampin for NTM-PD. However, adverse effects should be closely monitored when using rifabutin to replace rifampicin.

We analyzed the MIC profile changes in 55 patients with persistent culture positivity at treatment month 6. The DST profiles of clarithromycin and amikacin changed from susceptible to resistant and from resistant to susceptible. However, the MIC values of moxifloxacin and ethambutol only showed minor changes. A series of studies found that reinfection occurs more often than true relapse in MAC pulmonary disease (45 – 47). In the present study, basic analyses of genotypes for the isolates tested for drug susceptibility twice were not performed. Therefore, it is unclear whether the changes in DST profiles were due to recurrence or reinfection. Further studies are needed to clarify this issue.

The present study has several limitations. First, this was a retrospective, single-center study with a relatively small number of patients. Although the findings require verification in a larger study, the risk and protective factors identified in this study are helpful for clinical decision-making. Second, MIC values were determined on a single occasion due to the consideration of cost in clinical practice. Despite the inherent variations that exist for this microdilution method, the changes in drug-resistance profile presented in this study were at least fourfold dilutions, which cannot be simply attributed to method variations. Third, whole-genome sequencing was not performed to exclude the possibility of reinfection during antimycobacterial treatment. This makes it challenging to explain the changes in drug- resistance profiles; for instance, it is unclear why the DST changed from clarithromycin resistant to clarithromycin susceptible while patients were receiving less than the standard dose of clarithromycin.

### Conclusions

A higher risk of failed culture conversion by month 6 was identified in patients with a current smoking status, previous TB treatment, chronic lung disease, positive AFB smear before treatment, presence of cavitary lesions, adverse drug reactions, and clarithromycin resistance. Furthermore, the use of ethambutol, the number of effective drugs, and the absolute lymphocyte count at the initiation of treatment play an important role in the prognosis of *M. intracellulare* pulmonary disease. The changes in MIC values during antimycobacterial treatment indicated the necessity of monitoring to enable the timely adjustment of the treatment regimen.

## MATERIALS AND METHODS

### Study design and participants

This retrospective case-control study included patients newly diagnosed with *M. intracellulare* pulmonary disease between April 2017 and September 2021 in Shanghai Pulmonary Hospital. The criteria for diagnosis were in accordance with the 2007 ATS/IDSA guidelines (15). The positive group was defined as patients who failed to achieve sputum culture conversion after 6 months of antimycobacterial treatment. A previous study has indicated that a lack of microbiological response after 6 months of treatment is well predictive of culture conversion failure at 12 months (48), and it had been used as an important interim endpoint of efficacy in recent clinical trials (49, 50). The negative group consisted of patients who achieved sputum culture conversion or failed to expectorate sputum but had a stable radiological presentation at 6 months and were matched in a 1:1 ratio to the positive group by age and sex (51).

The design of the initial regimen generally referred to the ATS/ERS/ESCMID/IDSA guidelines from 2007 (15), with a macrolide, a rifamycin, ethambutol, and/or amikacin as the cornerstone if no clarithromycin resistance is identified for the baseline isolate. The variation of initial regimens mainly arises from the following aspects: (i) amikacin or fluoroquinolones will be added to the regimen if clarithromycin resistance is identified; (ii) patients with FC disease or severe nodular/bronchiectatic disease will be given parenteral amikacin as well for the first 2–3 months of therapy; (iii) some physicians in China prefer to add a fluoroquinolone into the regimen based on their clinical experience. Daily drug dosages are based on the patient’s weight as recommended by the ATS guidelines (15). After the initiation of treatment, the regimen might be changed for patients with adverse drug reactions, most of which are seen in the first few weeks. Then, the regimen will be relatively stable.

In routine clinical practice, patients with *M. intracellulare* pulmonary disease are regularly followed up every 3 months during antimycobacterial treatment. At each visit, sputum samples, if available, are collected for microscopy and culture. Blood samples are collected to monitor the renal and liver functions to detect drug-induced adverse effects. Computed tomography (CT) is performed to reveal the response to antimycobacterial treatment. In the present study, all baseline strains for both groups and the 6-month follow-up strains for the positive group were re-cultured for MIC testing. Medical charts were reviewed to extract the demographic characteristics, clinical features, radiographic findings, and laboratory test results.

### Main definitions

Microbiological persistence was defined as the continuous presence of culture positivity for *M. intracellulare* in respiratory samples after antibiotic treatment. Patients with failure to expectorate sputum but with stable radiological presentation at 6 months were considered to have achieved sputum culture conversion (51). Sputum culture conversion was defined as at least three consecutive negative sputum cultures collected at least 4 weeks apart (52). Radiological presentations were classified as FC, C-NB, NC-NB, and unclassifiable forms (45). The treatment regimen was defined as the one used for majority of the time in the first 6 months. In accordance with the ATS/ERS/ESCMID/IDSA clinical practice guidelines (10), apart from fluoroquinolones, other antibiotics commonly used in MAC treatment were counted as effective drugs, mainly clarithromycin, ethambutol, rifampin, rifabutin, and amikacin.

### Species identification and MIC determination

All isolates were collected from patients with *M. intracellulare* pulmonary disease between April 2017 and September 2021 in Shanghai Pulmonary Hospital. Isolates were grown on either liquid medium (BACTEC MGIT 960) or solid medium (Löwenstein–Jensen). All isolates were identified as NTM by the conventional method with para-nitrobenzoic acid and thiophene-2-carboxylic acid hydrazide in solid media (53). Additionally, the species of the isolates were identified using MeltPro Mycobacteria Identification Kit (ZEESAN Biotech, Xiamen, China), a licensed commercially available kit for the identification of 19 common *Mycobacterium* species, based on the probe-based melting curve analysis.

MIC determination was performed for the freshly re-cultured strains using a commercial SLOMYCO Sensititre MIC Plate (Trek Diagnostic System, Thermo Fisher, USA). In brief, appropriate amounts of bacterial strains were grown on the Löwenstein–Jensen medium before being subjected to DST. Using an ultrasonic grinder (TB Healthcare, China), 0.5 McFarland bacterial suspensions were prepared from the colonies grown on Löwenstein–Jensen medium, and 50 mL of bacterial suspension was added to 10 mL of Middlebrook Mueller-Hinton broth (Becton Dickson) to obtain a final concentration of 10^5^ CFU/mL and used for the DST. The diluted bacterial solution (100 µL/well) was distributed into each well using an automatic dispenser and incubated at 37°C for 7 days, and the growth of positive-control wells was observed. In case of poor growth in the positive-control well at 7 days, the plates were re-incubated and read at 10 or 14 days depending on its growth. *M. avium* ATCC700898 was used as quality control. The following 13 drugs were tested in accordance with the manufacturer’s protocols: clarithromycin, rifabutin, ethambutol, isoniazid, moxifloxacin, rifampin, trimethoprim/sulfamethoxazole, amikacin, linezolid, ciprofloxacin, streptomycin, doxycycline, and ethionamide. The MIC was determined as the lowest concentration of an antimicrobial agent that prevented the visible growth of a microorganism in a broth dilution susceptibility test. The breakpoints to define susceptibility and resistance were based on the CLSI guidelines (30). The tested concentration ranges of the 13 antibiotics by broth microdilution ranges are shown in Table 4.

**TABLE 4:** Broth microdilution range (mg/L) for 13 antimycobacterial drugs in this study

Antimicrobial agent	Broth dilution range (mg/L)
Amikacin	1–64
Ciprofloxacin	0.12–16
Clarithromycin	0.06–64
Doxycycline	0.12–16
Ethambutol	0.5–16
Linezolid	1–64
Moxifloxacin	0.12–8
Rifampin	0.12–8
Rifabutin	0.25–8
Trimethoprim/sulfamethoxazole	0.12–8
Streptomycin	0.5–64
Isoniazid	0.25–8
Ethionamide	0.3–20

### Statistical analysis

All data are shown as proportions for categorical variables; as mean ± standard deviation (SD) for normally distributed data; or as median with interquartile (IQR) for nonnormally distributed continuous variables. Chi-square or Fisher’s exact test, or Wilcoxon rank-sum test, were used for between-group comparisons, as appropriate. Considering the matched case-control design, conditional logistic regression was used to identify the risk factors for persistent positive culture after 6-month antimycobacterial treatment. Statistical significance was considered when the 95% CI of the odds ratio did not include 1. The minimum inhibitory concentrations were compared between baseline and 6-month clinical isolates, if available, in those taking corresponding drugs using repeated measures analysis of variances (ANOVA). *P* values < 0.05 were considered statistically significant. All analyses were performed with IBM SPSS 26.0 (IBM Corp., Armonk, NY).

## Data Availability

The dataset supporting the conclusions of this article is available from the corresponding authors on reasonable request.
